# Outcomes of 45 μm gelatin stent surgery over 24‐month follow‐up

**DOI:** 10.1111/ceo.14181

**Published:** 2022-11-06

**Authors:** Aleksandar Vukmirovic, Jessica Ong, Aqif Mukhtar, Dao‐Yi Yu, William H. Morgan

**Affiliations:** ^1^ Lions Eye Institute Perth Western Australia Australia; ^2^ Ophthalmology department Royal Perth Hospital Perth Western Australia Australia; ^3^ Centre for Ophthalmology and Visual Science University of Western Australia Perth Western Australia Australia

**Keywords:** gelatin stent, glaucoma, intraocular pressure, microfistula, XEN

## Abstract

**Background:**

The main objectives of this study were to determine whether known risk factors for trabeculectomy failure similarly influence gelatin stent outcomes and to identify surgical factors which may optimise success.

**Methods:**

A retrospective, observational study was conducted at a single centre in Perth, Western Australia over 24 months. Two‐hundred and sixty‐two eyes of 207 patients underwent XEN‐45 stent surgery with various forms of glaucoma. Surgical and postoperative data on subjects undergoing XEN‐45 stent surgery was collated. Intraocular pressure (IOP) reduction success was determined using three criteria: 1; IOP <18 mm Hg, 2: IOP <15 mm Hg and 3: >25% IOP reduction from baseline. Kaplan–Meier, mixed effects Cox Proportional hazard model and Chi‐Square test were used to measure survival of functioning stents.

**Results:**

The success rates at a maximum of 2 years after surgery by criteria 1, 2 and 3 were 61.3%, 26.2% and 28.9% in primary open angle glaucoma (*n* = 243), 18.8%, 16.9%, 21.4% in angle closure glaucoma (*n* = 11), 0%, 0%, 66.7% in congenital glaucoma (*n* = 5) and 0% in uveitic glaucoma (*n* = 3). No significant reduction in success was found in those eyes that had prior ocular surgery (all *p* > 0.07).

**Conclusions:**

Prior cataract or trabeculectomy surgery does not appear to adversely affect gelatin stent outcomes over 2 years follow up. Gelatin stent surgery appears to have less IOP reduction effect compared to trabeculectomy at 2 years.

## 1 INTRODUCTION

Gelatin stent surgery is a method of creating a channel between the anterior chamber (AC) and conjunctival tissue similar to trabeculectomy without conjunctival dissection.^1^ Trabeculectomy has long been considered the gold standard in glaucoma surgery since it was described by Cairns over 50 years ago.^2^ The survival rate of trabeculectomy after 20 years is approximately 60% without topical medication and 90% with topical medication.^3^ Published studies report up to 20%–40% of patients fail to maintain their target intraocular pressure (IOP) with trabeculectomy alone within 1 year of surgery resulting in the recommencement of medical therapy, additional interventions or further surgery.^4^, ^5^, ^6^

It is well known that there are several major risk factors for early failure of trabeculectomy which include age, ethnicity, type of glaucoma, previous trabeculectomy or cataract surgery and number of preoperative topical medications used.^3^, ^7^, ^8^, ^9^ Patients with primary open angle glaucoma (POAG) tend to have better outcomes with trabeculectomy compared to those with uveitic glaucoma.^3^ Repeat trabeculectomies generally have worse survival than initial trabeculectomies.^8^, ^9^ Caucasian eyes tend to have greater IOP reduction after trabeculectomy than non‐Caucasian eyes.^10^

Trabeculectomy surgery involves creating an artificial route for aqueous humour to flow into the subconjunctival space to achieve a lower IOP.^11^ Gelatin stents create a channel allowing subconjunctival filtration. Our study examines medium term outcomes of a particular cross‐linked gelatin stent commercialised as XEN (Allergan, Inc., Irvine, CA). Our aims were to establish whether risk factors known to influence trabeculectomy survival similarly applied to gelatin stent outcomes and to identify surgical variables which may improve surgical success.

## 2 METHODS

This work conformed to the Declaration of Helsinki and was conducted with approvals from the Human Ethics Committee of the University of Western Australia (Ref: RA/4/20/5284).

### 2.1 Study design

This was a retrospective, non‐randomised clinical study. It was conducted at a single centre, the Lions Eye Institute in Perth, Western Australia. All gelatin stent surgeries were performed by a single surgeon.

Inclusion criteria was any patient over the age of 18 years with primary open angle or angle closure glaucoma (ACG), primary congenital glaucoma or secondary glaucoma including pseudoexfoliative glaucoma and uveitic glaucoma who had gelatin stent surgery. ACG was defined as eyes that had primary angle closure with iris to trabecular meshwork contact with or without synechiae formation and who had laser iridotomy or cataract extraction with resultant significant angle opening. Gonioscopy was performed on all subjects to confirm partial or total angle opening with no signs of ciliary block to ensure these eyes were suitable for gelatin stent implantation. Exclusion criteria included those eyes that had not undergone gelatin stent surgery. It is worth noting that no subjects with totally occluded angles underwent gelatin stent surgery and so they were excluded. The site of gelatin stent insertion was always through a region of angle that was open. There were no patients with pigmentary glaucoma in our retrospective cohort and so this subtype was not included. Our cohort included only a very small number of patients with pseudoexfoliation and so these eyes were incorporated into the POAG group. Gelatin stents were implanted in eyes that demonstrated glaucomatous progression despite maximum tolerated medical therapy. Progression criteria was based on retinal nerve fibre layer loss demonstrated on serial optical coherence tomography (OCT) scans and/or worsening visual field loss consistent with glaucomatous progression.

There was no baseline IOP cut‐off for inclusion. Patients with a prior history of glaucoma filtration surgery (e.g., trabeculectomy) or cataract surgery were included. Patients with neovascular glaucoma were excluded. There was no medication washout period prior to surgery and patients remained on their regular glaucoma medication until the day of surgery.

All patients were diagnosed with glaucomatous optic neuropathy by a glaucoma subspecialist following a comprehensive evaluation with clinical examination, standardised automated perimetry (24‐4 Humphrey field analyser) and OCT. During preoperative assessment, patients were required to have healthy, mobile conjunctiva at the anticipated filtration bleb site and an open angle with visible trabecular meshwork.

The primary endpoint of this study was to examine the effect of key baseline factors on gelatin stent survival with regards to IOP reduction across a medium‐term follow up period of 24 months. Secondary endpoints included number of postoperative glaucoma medications, rate of needling with 5‐fluorouracil (5‐FU) and postoperative complications.

### 2.2 Surgical technique

All surgeries were performed using a standardised technique in an operating theatre. Surgical implantation was routinely performed under local anaesthesia with a combination of oxybuprocaine hydrochloride 0.4% w/v and subconjunctival lignocaine 2% injection. Patients received either no mitomycin C (MMC), a low dose of 5 μg (0.005 mg per 1 ml) or a high dose of 37.5 μg (0.0375 mg per 1 ml) of MMC intraoperatively. At the commencement of the study, five subjects were not given MMC to avoid MMC‐related toxicity as preoperative assessment revealed thin, fragile conjunctiva. However, bleb fibrosis developed rapidly postoperatively and the decision was made to introduce the use of MMC at a standard dose (37.5 μg). After the first year of the study, the Allergan advisory committee reviewed the MMC dosage and reached a consensus to use a lower metered dosage (5 μg) to compare the safety and efficacy to the higher dose. This timepoint served as a cut‐off whereby all patients prior to this received 37.5 μg MMC and all patients after one‐year received 5 μg, eliminating bias between these groups. MMC was injected subconjunctivally at the bleb site with a 30‐gauge needle prior to gelatin stent implantation.

A Yu ‐ Morgan (ASICO, Westmont, IL) speculum was used to stabilise the eye by fixating the inferotemporal limbus to an adjustable arm of the speculum with three 6‐0 silk sutures to provide counter‐traction during implantation. An inferotemporal clear corneal incision was made to introduce the needle of the preloaded device into the AC. This allowed the XEN‐45 gelatin stent to be placed superonasally. Stents were placed around the limbus at 1 and 2 o'clock in right eyes and 10 and 11 o'clock in left eyes. In a small number of eyes, superior placement was precluded due to subconjunctival scarring from previous ocular surgery or presence of an implant (e.g., scleral buckle) resulting in inferior placement of the gelatin stent. Sodium hyaluronate (ProVisc®) was injected to deepen the AC and improve visualisation of the angle. The introducer needle was passed across the AC, engaged the angle aiming for trabecular meshwork and advanced through the sclera until the needle tip emerged subconjunctivally approximately 3 mm posterior to the limbus. A mirrored goniolens was used to directly visualise the advancement of the needle through the angle. The gelatin stent was then delivered. The silk sutures were removed as well as the ProVisc® from the AC. The clear corneal incision was hydrated to achieve wound closure. At the Allergan advisory committee meeting at one‐year, it was decided to perform a comparative study of eyes that received depot steroid and those that did not. All eyes after this timepoint (121 eyes) were given depot steroid when available. Depot steroid was administered subconjunctivally (triamcinolone 40 mg/ml, Aspen Pharma, St Leonards, Australia) into the inferior fornix.

### 2.3 Postoperative management

Patients were instructed to cease all preoperative glaucoma medication in the operated eye on the day of surgery. Patients were routinely evaluated postoperatively at day 1; 1 and 4 weeks; and 2, 3, 6, 9, and 12 months. A standard postoperative drop regime was commenced the day after surgery consisting of prednisolone acetate (1%)/phenylephrine hydrochloride (0.12%) (Allergan, North Sydney, Australia) six times a day and ketorolac tromethamine 0.5% (Allergan, North Sydney, Australia) and chloramphenicol 0.5% (Aspen Pharma, St Leonards, Australia) four times a day. Chloramphenicol was ceased 2 weeks after surgery. Prednisolone/phenylephrine was reduced to four times a day after 1 month and continued with Ketorolac four times a day for 3 months. Patients who had previous cataract surgery were commenced on Fluorometholone 0.1% eye drops (Allergan, North Sydney, Australia) once daily indefinitely after the 3 months.

Bleb needling was performed at the discretion of the surgeon when bleb fibrosis/failure was observed. Needling was performed at the slit lamp under topical anaesthesia with oxybuprocaine hydrochloride 0.4% w/v using a 26‐gauge needling to dissect fibrous tissue followed by the injection of 5‐FU (50 mg/ml). Post‐needling, patients were prescribed prednisolone acetate (1%)/phenylephrine hydrochloride (0.12%) and chloramphenicol 0.5% (Chlorsig®) four times a day for 2 weeks.

### 2.4 Data collection

Data were gathered from a specialised database created using Microsoft Access (Microsoft Corporation, Redmond, WA, United States) used to record clinical information. Information up to 24 months after surgery was extracted and analysed. This included IOP at each visit, ethnicity, type of glaucoma, site of entry of gelatin stent in the irodocorneal angle (based on gonioscopic examination), 5‐FU needling and complications. Preoperative IOP was defined as the IOP recorded within 40 days prior to surgery. If there were two or more visits in this period, an average of the IOP measurements was calculated and taken to be the preoperative IOP.

### 2.5 Statistical analysis

Data were extracted from the database and converted to a CSV spreadsheet and imported into R (version 4.0.3) statistical software for data analysis. IOP trends were analysed by calculating the mean, standard deviation, median and range values. Results were described as means and standard deviations as well as counts and percentages. ANOVA analysis (multivariate) was used to compare mean IOP and numbers of medications between subgroups at various time points. A *p*‐value of <0.05 was considered statistically significant.

Chi‐Square test was performed to analyse the frequency of 5‐FU needling postoperatively and the frequency of complications in various subgroups. The effectiveness of the gelatin stent was assessed using Kaplan–Meier survival analysis. Three criteria for success were used for this study. Criterion 1 IOP less than 18 mm Hg, criterion 2 IOP less than 15 mm Hg and criterion 3 > 25% IOP reduction from baseline with or without medications. Impacts of baseline factors on success of gelatin stent survival were identified using the mixed effects Cox Proportional hazard model for both univariate and multivariate analysis. A Mixed effects model was used to deal with possible patient, intereye and surgical dates correlating within a subject. Variables including age, sex, previous cataract surgery, previous trabeculectomy surgery, depot steroids, number of preoperative medications, number of postoperative medications, ethnicity, needling rates, MMC dose, glaucoma diagnosis and sites of gelatin stent entry were first evaluated using a univariate model and then followed by a multivariate model analysis. A Linear mixed Poisson model was also utilised to analyse significance in factors associated with 5‐FU needling.

Given that three criteria were examined and the same dataset was used to test each criterion there is a risk of false discovery of null hypotheses by performing multiple comparisons. To manage this risk we used the Benjamini‐Hochberg procedure which ranks *p*‐values from each criterion against a step‐wise modified alpha (0.0167, 0.0333, 0.05) given three comparisons.^12^

## 3 RESULTS

A total of 262 eyes of 207 patients who underwent gelatin stent implantation were analysed over a 24‐month period with a mean baseline IOP of 20.40 mm Hg. A complete description of the baseline demographics and ocular characteristics can be found in Table 1.

**TABLE 1:** Baseline factors of the study participants

Age	69.9 ± 12.8
Female (eyes) sex	165 (63.0%)
Types of Glaucoma	
POAG	243 (92.7%)
ACG	11 (3.9%)
Primary congenital glaucoma	5 (1.9%)
Uveitic glaucoma	3 (1.08%)
Mitomycin C dose	
Nil	5 (1.9%)
5 μg	101 (38.6%)
37.5 μg	156 (59.5%)
History of cataract surgery	66 (23.7%)
History of trabeculectomy surgery	47 (15.3%)
No. medication at baseline (*n* = 170)	2.70 ± 1.01
Baseline IOP	20.40 ± 6.31

The mean number of medications used reduced from 2.70 (±1.01, *n* = 170) preoperatively to 0.33 ± 0.78 at 3 months (*n* = 214), 0.54 ± 0.92 at 12 months (*n* = 221) and 0.60 ± 0.96 after 2 years of treatment (*n* = 221).

**FIGURE 1:**
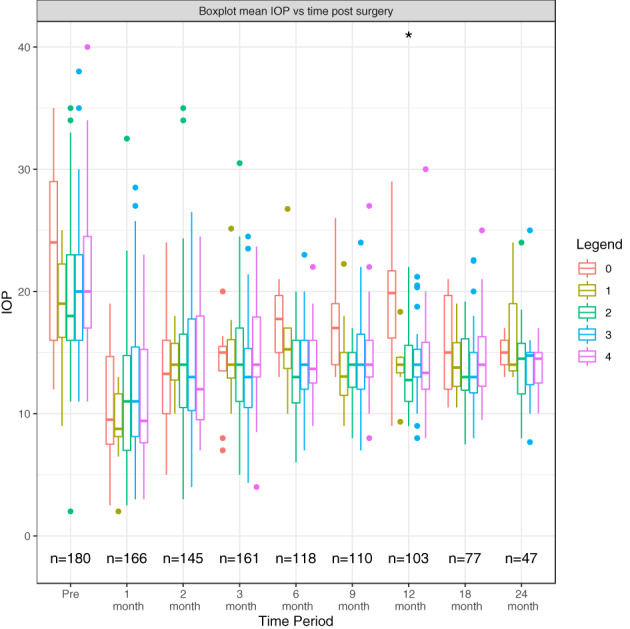
Boxplot illustrating mean IOP among eyes based on preoperative medication usage following surgery over 24 months. *Eighty‐two patients were omitted as they had no recorded pre medication usage data.

IOP post‐surgery was not significantly influenced by preoperative medication usage with a significance only found at 12 months post‐surgery (*p* = 0.0271) but at no other time point over 24 months (Figure 1). The numbers of subjects in each group varied greatly: 0 medications (*n* = 9), 1 medication class (*n* = 14), 2 medication classes (*n* = 55), 3 medication classes (*n* = 59) and 4 medication classes (*n* = 43).

**FIGURE 2:**
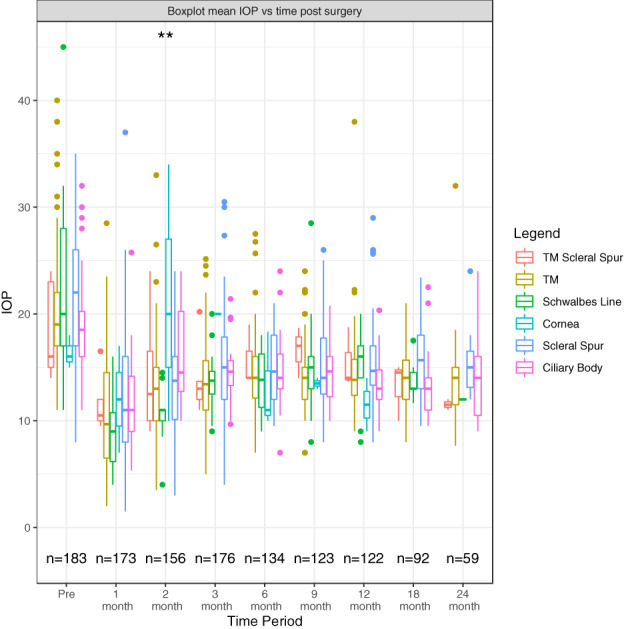
Boxplot illustrating mean IOP among eyes based on gelatin stent site of entry following surgery over 24 months. *Seventy‐nine patients were omitted as they either had no recorded site of entry data or an unknown site of entry.

The gelatin stent entry points analysed were trabecular meshwork (TM)/scleral spur junction (50.3%, *n* = 92), Schwalbes line (9.3%, *n* = 17), cornea (1.6%, *n* = 3), scleral spur (25.7%, *n* = 47) and ciliary body (13.1%, *n* = 24) (Figure 2). Gelatin stent position had no significant association with IOP at any postoperative time point except at 2 months post‐surgery (*p* = 0.009).

There was no significant difference between preoperative medication in eyes without prior ocular surgery (2.63 ± 1.03), prior cataract surgery (2.71 ± 1.23) or trabeculectomy (3.05 ± 0.84, min *p* = 0.13). There was no significant difference between postoperative medication in eyes without prior ocular surgery at 12 (0.47 ± 0.87 no prior surgery, 0.88 ± 1.13 prior trabeculectomy, 0.79 ± 1.17 prior cataract surgery) and 24 months (0.55 ± 0.91 no prior surgery, 0.76 ± 1.01 prior trabeculectomy, 0.79 ± 1.19 prior cataract surgery).

One hundred and eleven (42.4%) eyes required at least one bleb needling with 5‐FU within the first 12 months. In these eyes, the mean number of needlings was 1.78 (±1.0). Twenty‐eight (42.4%) eyes who had prior cataract surgery (*n* = 66) and 24 (51.1%) eyes who had prior trabeculectomy (*n* = 47) required at least one needling during the first 12 months after surgery. The mean needling rates in these eyes were 2.00 (±1.12) in the cataract surgery group and 2.12 (±1.36) in the trabeculectomy group.

A Poisson distributed generalised linear mixed model was used to analyse the effect of gelatin stent surgery on 5‐FU needling post gelatin stent surgery. The Poisson model found that prior cataract (*p* = 0.79), medication usage (*p* = 0.79) and IOP reduction (*p* = 0.27) were not associated with 5‐FU needling after surgery. There appeared to be an increased use of 5FU needling in subjects who had undergone prior trabeculectomy (*p* = 0.08, *c* = 0.5623 5‐FU) but it did not reach formal statistical significance.

Multivariate mixed effects Cox proportional analysis on all three IOP success criteria found the following factors had elevated hazard ratios and were significantly associated with failure. By criterion 1, uveitic glaucoma (*p* = 0.02) and no MMC (*p* = 0.03) had higher rates of failure. By criterion 2 failure was increased with no MMC (*p* = 0.02). By criterion 3, uveitic glaucoma (*p* = 0.001) and reduced postoperative medication usage (*p* = 0.007) were associated with greater failure. By criterion 1 angle closure glaucoma (*p* = 0.03) appeared to have increased failure rates but this did not reach formal statistical significance using the Benjamini‐Hochberg ranked probability adjustment. No significant reduction in success was found in those eyes that had prior ocular surgery (all *p* > 0.07). Association between all multivariate factors by all criterions can be seen in Table 2.

**TABLE 2:** Univariate and multivariate hazard models

Variables	Criterion 1		Criterion 2		Criterion 3	
	Hazard ratio	*p*	Hazard ratio	*p*	Hazard ratio	*p*
**Univariate**						
Age	1.00	0.61	1.00	0.68	1.01	0.32
Sex (male)	1.09	0.71	0.84	0.33	0.75	0.12
Prior surgery (compared to no prior surgery)						
Prior cataract	1.35	0.31	1.12	0.63	1.20	0.43
Prior						
Trabeculectomy	0.61	0.40	1.14	0.73	1.36	0.40
Prior						
Trabeculectomy and cataract	1.36	0.42	0.85	0.63	1.44	0.22
Depot steroids	1.15	0.56	1.01	0.98	1.02	0.93
No. preoperative medications	1.05	0.70	0.95	0.58	1.00	0.96
No. postoperative medications	1.32	0.02*	1.10	0.38	0.68	0.02*
Ethnicity (Caucasian)	1.00	0.98	0.71	0.16	0.93	0.75
Needling	1.02	0.86	1.07	0.47	0.86	0.19
Mitomycin C dose (compared to 37.5 μg)						
Nil	2.99	0.04	2.61	0.04	1.41	0.63
5 μg	1.43	0.15	1.20	0.32	1.06	0.74
Types of Glaucoma (compared to POAG)						
ACG	3.70	0.002**	1.72	0.16	1.25	0.62
Congenital	7.45	0.001***	3.08	0.03*	0.84	0.87
Uveitic	5.70	0.02*	2.49	0.20	7.46	0.006**
Site of entry (compared to TM)						
Schwalbes line	0.29	0.09	0.59	0.17	0.74	0.41
Cornea	2.52	0.21	1.25	0.76	1.02	0.97
Scleral spur	1.09	0.75	1.27	0.26	0.72	0.18
Ciliary body	0.84	0.58	1.06	0.84	1.28	0.35
**Multivariate**						
No. postoperative medications	1.13	0.43	0.99	0.95	0.58	0.007**
Mitomycin C dose (compared to 37.5 μg)						
Nil	4.93	0.03*	4.11	0.02*	2.38	0.27
5 μg	2.26	0.10	2.03	0.06	1.16	0.71
Types of Glaucoma (compared to POAG)						
ACG	3.83	0.03	1.94	0.20	1.66	0.36
Congenital	2.32	0.45	2.08	0.35	3.65	0.26
Uveitic	6.47	0.02*	2.94	0.17	12.62	0.001**

* *p* < 0.05;

** *p* < 0.01.

*** *p* < 0.001.

**FIGURE 3:**
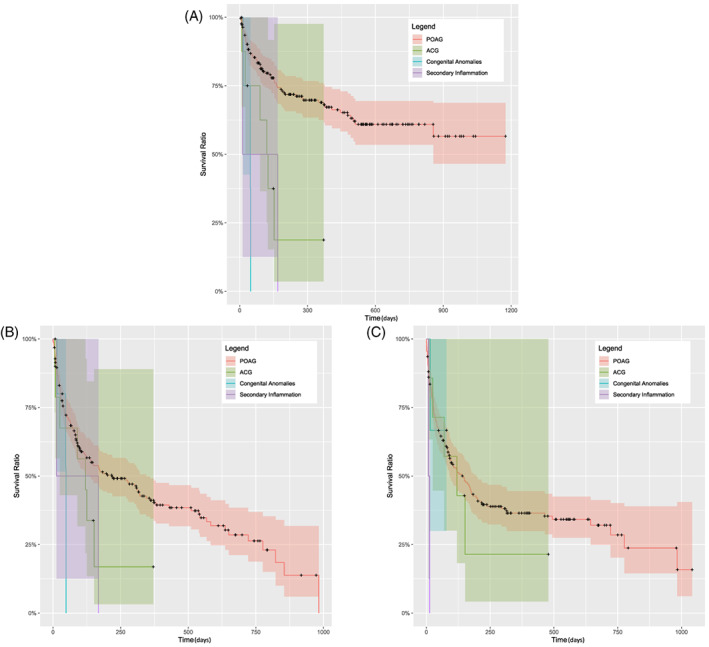
Kaplan–Meier survival cases by glaucoma diagnosis subgroup (A–C) by criterion 1 (A), by criterion 2 (B), by criterion 3 (C). Solid lines show the cumulative probability of success and the shaded areas show the 95% confidence intervals. POAG indicates primary open angle glaucoma and ACG indicates angle closure glaucoma.

**FIGURE 4:**
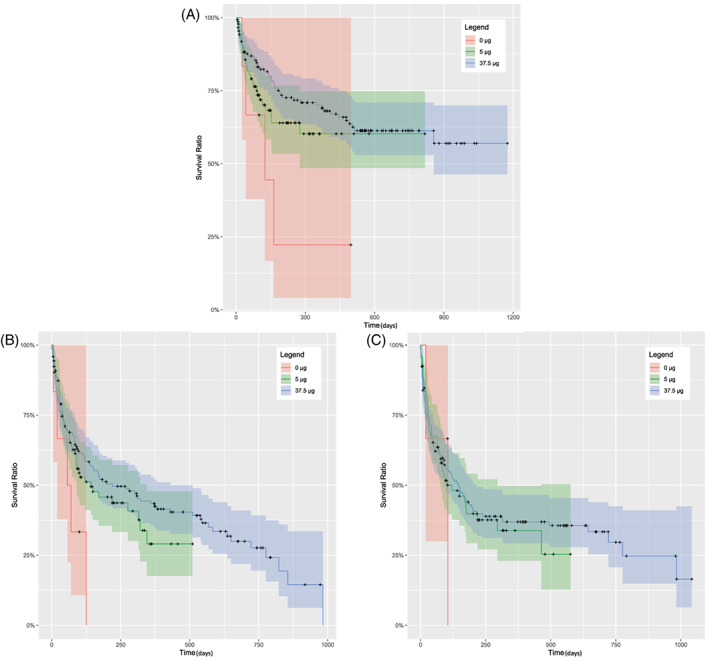
Kaplan–Meier survival cases by mitomycin C subgroup (A–C) by criterion 1 (A), by criterion 2 (B), by criterion 3 (C). Solid lines show the cumulative probability of success and the shaded areas show the 95% confidence intervals.

By criterion 1, probability of survival at 2 years post‐surgery was 61.3% in POAG, 18.8% in ACG (350 days post‐surgery), 0% in congenital glaucoma and uveitic glaucoma (Figure 3). Cumulative probability of survival at 2 years post‐surgery was 61.3% with 37.5 μg MMC dose, 61.1% with 5 μg MMC dose and 22.2% (500 days post‐surgery) with no MMC (Figure 4, Table 2).

By criterion 2, probability of survival at 2 years post‐surgery was 26.2% in POAG, 16.9% (375 days post‐surgery) in ACG, 0% in congenital glaucoma and uveitic glaucoma (Figure 3). The probability of survival at 2 years post‐surgery using various MMC doses varied from 27.7% in 37.5 μg MMC dose, 28.4% (500 days post‐surgery) in 5 μg MMC dose and 0% with no MMC (Figure 4, Table 2).

By criterion 3, glaucoma diagnosis cumulative probability of survival at 2 years post‐surgery was 28.9% in POAG, 21.4% (490 days post‐surgery) in ACG, 66.7% (75 days post‐surgery) in congenital glaucoma and 0% in uveitic glaucoma (Figure 3). MMC dosage cumulative probability of survival at 2 years post‐surgery was 29.7% with 37.5 μg MMC dose, 28.3% (575 days post‐surgery) with 5 μg MMC dose and 0% with no MMC (Figure 4, Table 2).

We were interested in examining if there was a relationship between MMC dosage and depot steroids in regard to gelatin stent failure. Mixed effects Cox proportional analysis on all three criteria found the following factors associated with failure. By criterion 1, 2 and 3 no significance was found between 37.5 μg MMC dose with depot steroids, 37.5 μg MMC dose without depot steroids, 5 μg MMC dose with depot steroids and 5 μg MMC dose without depot steroids (All *p* > 0.22). Gelatin stent surgery without MMC by criterion 1 (*p* = 0.022) and 2 (*p* = 0.012) was found to be associated with a higher likelihood of gelatin stent failure. However, no association was found in criterion 3 between zero dose of MMC and gelatin stent failure.

The complications and their frequency are described in Table 2. Prior surgery (Table 3) and MMC dosage (Table 4) were found not to affect the rates of complications in our study (all *p* value >0.17). The frequency of 5‐FU needling rates was also analysed (Table 5) and no association was found between needling rates of eyes with and without prior ocular surgery (minimum *p* = 0.18).

**TABLE 3:** Complication rates between eyes having gelatin stent surgery with and without prior ocular surgery

	Overall (%)	No prior trabeculectomy (%)	Prior trabeculectomy (%)	*Trabeculectomy p* value	No prior cataract (%)	Prior cataract (%)	*Cataract p* value
Hyphaema	15.6	15.8	14.7	0.87	15.7	15.5	0.98
Dellen	3.1	3.5	0	0.27	3.9	3.4	0.87
Hypotony with shallow anterior chamber	6.1	6.6	2.9	0.41	5.9	6.9	0.78
Hypotony viscoelastic fill	0.4	0.4	0	0.70	0.5	0	0.59
Choroidal effusions	3.8	4.4	0	0.21	2.9	6.9	0.17

**TABLE 4:** Complication rates between eyes having gelatin stent surgery and receiving different doses of mitomycin C

	Overall (%)	5 μg (%)	37.5 μg (%)	*p* Value
Hyphaema	16.0	15.8	16.0	0.97
Dellen	3.1	2.0	3.8	0.40
Hypotony with shallow anterior chamber	6.2	5.9	6.4	0.88
Hypotony viscoelastic fill	0.4	0	0.4	0.42
Choroidal effusions	3.9	4.0	3.8	0.96

**TABLE 5:** Frequency of 5‐FU needling rates among eyes with and without prior ocular surgery

	Overall (%)	No prior trabeculectomy (%)	Prior trabeculectomy (%)	*Trabeculectomy p* value	No prior cataract (%)	Prior cataract (%)	*Cataract p* value
Needling rate	42.4	40.5	51.1	0.18	42.3	42.4	0.99

## 4 DISCUSSION

Surgery in POAG eyes with the XEN‐45 gelatin stent in our hands had a survival rate of 61% for IOP < 18 and 26% for IOP < 15 at 2 years. This was inferior to the trabeculectomy results from Coleman^13^ finding a 3‐year survival of 61% for IOP < 18 with 20% IOP reduction and 52% for IOP < 15 and 25% IOP reduction. Our 2 years 25% IOP reduction survival for POAG subjects was 29%. The ability of XEN‐45 surgery to achieve IOP less than 15 mmHg or long term 25% IOP reduction appears limited by comparison to trabeculectomy.^9^, ^13^

We found gelatin stent induced pressure reduction over 2 years was generally not influenced by some risk factors recognised to adversely influence trabeculectomy outcomes.^3^, ^14^, ^15^ Our results demonstrated no statistically significant effect of ethnicity on gelatin stent survival. However, our dataset was biased comprised of a majority of Caucasian eyes (84%) and only a small number of South Asian, East Asian and African eyes. This precluded us from conducting subgroup analyses with meaningful results and so we are unable to make a confident statement regarding the effect of ethnicity on gelatin stent success rates.

Our study found that factors associated with failure were uveitic glaucoma and no MMC. There was a tendency towards failure in angle closure glaucoma which did not reach formal statistical significance. There are no published data comparing the efficacy of gelatin stents in POAG versus ACG and limited studies comparing trabeculectomy between these groups. Maheshwari et al. reported a greater mean total IOP reduction in the OAG group compared to ACG group in their study of 108 eyes at 3 years after trabeculectomy.^16^ However, Sihota et al. found no statistically significant difference in reduction of IOP to ≤20 mmHg without antiglaucoma medication between OAG and ACG at 10 years.^17^

Inflammation has been recognised to influence bleb failure. This may be related to wound healing where surgical trauma induces inflammation stimulating fibroblast proliferation and leading to subconjunctival fibrosis.^18^ Similarly, the pro‐inflammatory state of eyes with uveitic glaucoma would lead to higher bleb failure rates secondary to fibrosis. Thus, it was not surprising that eyes with no MMC had higher failure rates than eyes where antimetabolite was used to reduce bleb fibrosis.

We examined the effect of two different doses of intraoperative MMC. Our data using criterion 1 and 2 revealed a significantly increased likelihood of gelatin stent survival in eyes which received 37.5 μg MMC and 5 μg MMC compared to eyes which did not receive any MMC. We did not observe a difference in effect between and lower and higher MMC dose. There was no difference in rates of complications among eyes receiving different doses of MMC. The use of a lower dose of MMC may be desirable due to the risk of antimitotic toxicity and potential complications.

There have been mixed reports in the literature about the effect of the number and duration of topical glaucoma medications used preoperatively on bleb survival. Preoperative medication use has been associated with increased inflammatory mediators which have been found in the conjunctiva and Tenon's capsule of eyes receiving long‐term glaucoma medication.^3^, ^19^, ^20^, ^21^ It has been postulated that conjunctival inflammation induced by topical medications may be associated with higher rates of bleb failure however, there are conflicting results in the literature. Our study showed that preoperative medication usage had no significant adverse effect on postoperative IOP (Figure 1).

With univariate analysis, criterion 1 failure (IOP ≥ 18 mmHg) was associated with greater use of medications, whereas criterion 3 failure (IOP greater than 25% reduction) was associated with reduced use of medications. This latter observation was noted in the multivariate analysis also. This result likely reflects the tendency to increase medication use as IOP control is failing to maintain some IOP control (criterion 1) and also to augment successful drainage in subjects whose target IOP is low (criterion 3).

The risk of failure is higher in repeat trabeculectomy compared to primary trabeculectomy with generally inferior IOP control and a higher number of medications required to maintain target IOP.^3^, ^7^, ^13^, ^22^ Furthermore, studies have suggested that pseudophakic patients have higher rates of trabeculectomy failure than phakic patients.^8^, ^9^, ^10^ Our results demonstrated that eyes with prior trabeculectomy or cataract surgery had comparable gelatin stent survival and medication use compared with eyes with no prior surgery. To our knowledge, there is no data in the literature examining bleb survival when cataract surgery is performed after gelatin stent surgery. However, studies have compared combined phaco‐XEN to stand‐alone XEN surgeries and showed no significant difference in IOP reduction.^23^, ^24^ 5‐fluorouracil needling rates were not significantly different between subjects who had undergone prior trabeculectomy or not. We cannot explain why there is an apparently reduced impact of prior trabeculectomy or cataract surgery upon gelatin stent survival.

We investigated the effect of site of entry through the iridocorneal angle to determine whether this surgical variable could be modified to optimise outcomes. Our results revealed no significant effect of the site of entry on gelatin stent survival. This was not an unexpected result as the gelatin stent provides a channel between the AC and subconjunctival space by passing through the angle. It should be noted that very few stents were implanted through the cornea (*n* = 3) and so our results cannot be used to draw conclusions regarding corneal entry.

The complication rates in our largely Caucasian cohort appear favourable compared to the UK national survey of trabeculectomy complications.^25^ We found hyphaema occurred in 15.6% of eyes post gelatin stent implantation compared to 24.6% of eyes post trabeculectomy, hypotony with shallow AC occurred in 6.1% of eyes post gelatin stent implantation compared to 23.9% of eyes post trabeculectomy and choroidal effusions occurred in 3.8% of eyes post gelatin stent implantation compared to 14.1% of eyes post trabeculectomy.^17^ Most of these complications relate to bleb size and location (dellen) or hypotony (shallow AC and choroidal effusions). We had thought that the rates of hypotony would be lower than the first version of the gelatin stent having an internal diameter of 140 micron. However, this did not appear to be the case.^26^ We suspect that the large size of the introducer (300 micron) relative to the XEN outer diameter (90 micron) may allow peri‐stent leak in the postoperative period for a variable length of time. We suspect that avoiding excess production of secondary aqueous in the immediate postoperative period probably leads to reduced fibrosis and reduced need for antimitotics. Our earlier report on the 140‐micron gelatin stent outcomes came from patients who did not receive any antimitotics, some of whom had surgery without viscoelastic and had a 6‐year qualified success using criteria 1 of 78%. These stents had an outer diameter of 220 micron which with swelling may have blocked the needle track more effectively. Gelatin stent device technology has probably not reached maturity in terms of maximising the benefits of the gelatin stent biocompatibility and overall tissue flexibility.

One of the major limitations of this study was its retrospective, noncomparative design with small subgroup sample sizes particularly within the ethnicity and glaucoma subtype groups. This was a medium‐term study with positive results in favour of reduced risk factors for gelatin stent failure however, these results may change over a longer follow up period as surgical failure tends to increase over time. Additionally, the total number of eyes that had completed 24 months follow‐up was relatively small compared to those that had completed 12 months.

Our study highlights that many risk factors for trabeculectomy failure do not appear to influence gelatin stent outcomes over medium‐term follow up. However, XEN‐45 surgery appears to have less IOP reduction effect compared to trabeculectomy at 2 years.

## CONFLICT OF INTEREST

DY, YU and WH Morgan are inventors of original cross‐linked gelatine microfistula technology. WH Morgan has acted as an advisor for Allergan. WH Morgan has received a grant from Allergan to study medium term results of XEN45.
